# Modulation of cancer signalling pathway(s) in two -stage mouse skin tumorigenesis by annonacin

**DOI:** 10.1186/s12906-019-2650-1

**Published:** 2019-09-03

**Authors:** Mohd Rohaizad Md Roduan, Roslida Abd Hamid, Norhafizah Mohtarrudin

**Affiliations:** 1 Department of Biomedical Science, Faculty of Medicine and Health Sciences, Universiti Putra Malaysia, 43400 Serdang, Selangor Malaysia; 2 Department of Pathology, Faculty of Medicine and Health Sciences, Universiti Putra Malaysia, 43400 Serdang, Selangor Malaysia

**Keywords:** Annonacin, Antitumor promotion, Gene expression, Skin tumorigenesis

## Abstract

**Background:**

Annonacin, an annonaceous acetogenin isolated from *Annona muricata* has been reported to be strongly cytotoxic against various cell lines, in vitro. Nevertheless, its effect against in vivo tumor promoting activity has not been reported yet. Therefore, this study was aimed to investigate antitumor-promoting activity of annonacin via in vivo two-stage mouse skin tumorigenesis model and its molecular pathways involved.

**Methods:**

Mice were initiated with single dose of 7,12-dimethylbenz[α]anthracene (DMBA) (390 nmol/100 μL) followed by, in subsequent week, repeated promotion (twice weekly; 22 weeks) with 12-*O*-tetradecanoylphorbol-13-acetate (TPA) (1.7 nmol/100 μL). Annonacin (85 nM) and curcumin (10 mg/kg; reference) were, respectively, applied topically to DMBA/TPA-induced mice 30 min before each TPA application for 22 weeks. Upon termination, histopathological examination of skin, liver and kidney as well as genes and proteins expression analysis were conducted to elucidate the potential mechanism of annonacin.

**Results:**

With comparison to the carcinogen control, Annonacin significantly increased the tumor latency period and reduced the tumor incidence, tumor burden and tumor volume, respectively. In addition, it also suppressed tumorigenesis manifested by significant reduction of hyperkeratosis, dermal papillae and number of keratin pearls on skin tissues. Annonacin also appeared to be non-toxic to liver and kidney. Significant modulation of both AKT, ERK, mTOR, p38, PTEN and Src genes and proteins were also observed in annonacin-targeted signaling pathway(s) against tumorigenesis.

**Conclusions:**

Collectively, results of this study indicate that annonacin is a potential therapeutic compound targeting tumor promoting stage in skin tumorigenesis by modulating multiple gene and protein in cancer signaling pathways without apparent toxicity.

**Electronic supplementary material:**

The online version of this article (10.1186/s12906-019-2650-1) contains supplementary material, which is available to authorized users.

## Background

Chemotherapy plays an important role in the treatment of many cancer type including skin cancer. The chemical agents used in chemotherapy are selectively destructive to malignant cells, but these agents can also cause damage to the healthy normal cells, which results in adverse side effects that negatively impact compliance with cancer treatment, as well as its well-being [1]. Therefore, there is an imperative need to find a strategy to resolve the problem. This eventually has led to the discovery of new plant-based chemopreventive agent as an alternative strategy as plants-based medicines are generally considered safe when it is produced and used in an appropriate manner [2, 3].

Several evidences have demonstrated that *Annona muricata* L possesses pleiotropic effects such as anti-inflammatory, anticancer, antiparasitic, pesticidal, antimicrobial and antiviral activities [4–6]. Among these activities, anticancer activity has been intensively studied due to the exploration of cytotoxic compounds abundantly present in various parts of *Annona muricata* namely, annonaceous acetogenin [5–8].

Annonaceous acetogenins have been reported to exert cytotoxicity in various cancer cell lines such as lung, colon, pancreatic, prostate, and breast [5, 8]. For the past 30 years, several researchers have conducted numerous studies in elucidating the underlying mechanisms of actions of these compounds. Amongst the mechanisms reported are the inhibition of mitochondrial complex 1 enzyme, induction of apoptosis, blocking of the cell cycle through the halt at certain phases and inhibition of DNA topoisomerase [5, 7, 9, 10].

Annonacin, a mono-tetrahydrofuran acetogenin is one of the major compounds consists in *Annona muricata* leaf and seed and in other plants from Annonaceae family. It has been priorly reported to be actively cytotoxic against various cancer cell lines [5, 8–10]. Despite its role as a potent inhibitor of the mitochondrial complex I in the electron transport system, its other mechanisms in vivo, in tumorigenesis have yet to be extensively explored. To the best of our knowledge, the mechanisms of annonacin in modulating tumorigenesis pathways, in vitro, includes, upregulation of Bax and caspase-3 expression that lead to apoptosis [9]; inhibiting ER-α, cyclin D1 and Bcl-2 protein expressions [10]; inhibits HIF-1α and mTOR activation [11]; and downregulation of ERK protein expression [12]. These events eventually lead to cancer cell apoptosis.

Two-stage skin cancer model consists of multistage tumorigenesis, offers an investigational framework to study the basic mechanism linked with the initiation, promotion and progression stages in animal. It is also beneficial in examining chemopreventive agents that potentially affect any stages of tumorigenesis with regard to understand the molecular mechanism and evolution of cancer cells, not only in skin cancer, but in other multistage human cancers such as prostate and colon cancer [13]. Chemical-induced carcinogenesis occurs as a result of a complex multiple steps of event initiated by mutations via direct DNA damage coupled with an overproduction of reactive oxygen species [18]. A large number of evidence have demonstrated that the role of Ras/Raf/ERK and PI3K/AKT/mTOR pathways, *MAPK* (*p38*) and proto-oncogene (*Src*) as well as tumor suppressor gene (*PTEN*), in cell proliferation and apoptosis [19]. Upregulation of proteins in Ras/Raf/ERK and PI3K/AKT/mTOR pathways, *MAPK* (*p38*) and proto-oncogene (*Src*) as well as loss of tumor suppressor function (*PTEN*) have been implicated in several cancers including skin cancer [19, 20]. These signaling pathways impart cancer cells with neoplastic progression through uncontrolled cell proliferation and inhibition of apoptosis [21].

To date, all the mechanisms previously reported on annonaceous acetogenin including annonacin, were based on in vitro results. There is no study conducted in elucidating the mechanism of action of annonaceous acetogenin in any in vivo model yet. Thus, this current study was aimed to investigate the antitumor promoting effect and toxicity of annonacin, in two stage mouse skin tumorigenesis, as well as its molecular pathways in suppressing/inhibiting the skin tumorigenesis.

## Methods

### Chemicals and drugs

All chemicals used are of analytical grades unless otherwise specified. Annonacin was purchased from Progen Scientific (Cat No:CFN97856; 96% HPLC purity, isolated from *Annona muricata* leaves; London, UK), curcumin was purchased from Sigma-Aldrich (Cat No: 820354; > 95%; MO, USA), dimethylbenz[a]anthracene (DMBA) and 7, 12-O-tetradecanoylphorbol-13-acetate (TPA) were also purchased from Sigma-Aldrich (USA), acetone, hematoxylin and eosin (H&E) were purchased from Merck (Dramstaadt, Germany), phosphate buffer saline (PBS) was purchased from Dako (Glostrup, Denmark), ethanol, xylene and neutral buffer formalin were purchased from J.T Baker Chemicals (New Jersey, USA), Bio-Plex Pro Cell Signalling Assay Kit (Cat.No:LQ0-0000S6KL81S and LQ0-0006JK0K0RR), iScript cDNA synthesis kit (Cat.No: 4106228C), SsoAdvance universal SYBR green supermix kit (Cat.No: 1725271) were all purchased from Biorad (CA, USA), Nucleospin RNA-Protein extraction kit was purchased from Macherey-Nagel (Cat. No: 740933. Düren, Germany), RNA*later* was purchased from Life Science (Colorado, USA) and RNAse zap was purchased from Thermo Fisher Scientific (MA, USA).

### Experimental animals

50 female ICR mice purchased from local supplier (Sapphire Enterprise, Malaysia) with age between 6 and 7 weeks were used in this experiment. Ethical approval was obtained from the Institutional Animal Care and Use Committee (IACUC), Universiti Putra Malaysia prior to execution of the experiment. The reference number was UPM/IACUC/AUP-R068/2014 (Approval date: 14 January 2015). ICR mice with initial weight between 20 and 30 g were used throughout the experiment. These animals were housed at Animal Housing Facility, Faculty of Medicine and Health Sciences, Universiti Putra Malaysia. Mice were randomly separated into 5 groups (*n* = 10) in plastic cages contained wood shaving and fed with free access standard laboratory diet (food pellet and water) ad libitum. Animals were also maintained at temperature 25 ± 2 °C under 12-h light and dark cycle. Acclimatization was allowed for 1 week prior to commencement of the experiment. Mice were weighed before experimentation. All treatments were carried out at Animal Housing Facility and Pharmacology Laboratory, Faculty of Medicine and Health Sciences, Universiti Putra Malaysia. The dorsal of the mice were shaved approximately 2 cm × 2 cm with electric clipper 3 days before treatment.

### Anti-tumor promoting effects of annonacin (85 nM) in two-stage mouse skin tumorigenesis model

The experimental design was adapted from Abel et al. 2009 [13] as follows:

Group I (vehicle control): Mice were given topical application acetone (100 μL/mouse) on the shaved dorsal skin area twice weekly, throughout the experiment period.

Group II (carcinogen control): Mice were given single topical application of DMBA in acetone (390 nM/100 μL/mouse), followed by topical application of TPA in acetone (1.7 nM/100 μL/mouse) a week after DMBA application, twice weekly for 22 weeks of promotion period.

Group III (annonacin treatment): A week after DMBA single dose application, mice in this group were repeatedly promoted with TPA twice weekly for 22 weeks. 30 min prior to TPA application, mice were also applied topically with annonacin (85 nM/100 uL/mouse) twice weekly for 22 weeks.

Group IV (treatment control): Mice were given only annonacin (85 nM/100 uL/mouse) twice weekly for 22 weeks without DMBA initiation and TPA promotion.

Group V (reference control): Mice were treated as in Group III, except the animals received the topical application of curcumin (10 mg/kg/100 μL/mouse) instead of annonacin twice weekly for 22 weeks.

### Morphological assessment

During the period of induction and treatment, each mouse in all groups of the promotion stage was weighed and shaved weekly for an easy application of the carcinogens/test compound and skin lesion observation. Tumor latency, tumor incidence, tumor burden and tumor volume were observed, measured and recorded at weekly interval. Tumors with a diameter greater than 1 mm that persists for at least 2 consecutive observations were included in the cumulative counts. The data expressed as (i) tumor incidence (%) - ([number of mice with tumor/total number of mice] × 100%) [22]; (ii) tumor burden - (the total number of tumors per tumor-bearing mice) and; (iii) the tumor volume (mm^3^) = π/6 x length x width x height [23]. Whilst, (iv) the latency period of tumor formation was determined by the appearance of the first tumor and (v) tumor regression was determined by remission of established tumor [24].

### Histopathological assessment

All mice were sacrificed upon termination at week 22 of the experiment by cervical dislocation and sampled for further analysis. Treated mice skin area (with or without tumor) were harvested and rinse with PBS 1x. Part of harvested skin was preserved in RNA*later* (Life Science, USA) solution for molecular expression analysis. Whereas, the remaining specimens were preserved in 10% (v/v) neutral buffered formalin for standard hematoxylin and eosin (H&E) staining. The slides were evaluated by pathologist for assessment of the pathological changes and digital micrographs of the slides were taken using Dino-Lite microscope eyepiece camera (ANMO, Taiwan).

### RNA and protein extraction

Total RNA and protein from the skin tissues (with or without tumor) were extracted by using Nucleospin RNA and Protein extraction kit (Macherey Nagel, Germany). This kit employs parallel isolation of RNA and protein from undivided samples. Skin tissues were added into lysis buffer and then homogenized using tissue homogenizer (Qiagen, Germany). Homogenized tissues formed as a lysate were processed according to the protocol provided by the manufacturer accordingly to isolate RNA and protein separately. The eluted RNA and protein were kept at -80 °C and -20 °C for further analysis, respectively. The RNA concentration was determined using NanoDrop, ND-1000, (Thermo Scientific, USA), while the RNA integrity was evaluated via Bioanalyzer 2100 and an Agilent RNA 6000 Nano Kit (Agilent Technologies, USA).

### Quantification of gene expression via RT-qPCR

Single stranded cDNA (20 μL) was converted from RNA (1 μg) through reverse transcription by using iScript cDNA synthesis kit (Biorad, USA). The samples were incubated at 42 °C for 30 min and followed by 85 °C for 5 min. cDNA was hold at 4 °C for 2 min before being stored at − 20 °C. cDNA amplification of the target genes was performed using SsoAdvance universal SYBR green supermix kit (Biorad, USA) kit via standard protocol of quantitative real time-PCR. The amplicons used were synthesized and obtained commercially from Biorad, USA as follow: qHsaCID0011338 for *AKT* (143 bp), qHsaCID0006818 for *ERK1/2* (64 bp), qHsaCID0007341 for *p38* (61 bp), qHsaCED0048371 for *mTOR* (85 bp), qHsaCED0036796 for *PTEN* (117 bp) and qHsaCED0048158 for *Src* (107 bp). Also, *GAPDH* (qHsaCED0038674, 117 bp) and *GUSB* (qHsaCID0011706, 79 bp) genes were also amplified as internal controls (housekeeping genes) as these genes are constantly expressed in all tissues. All the amplicons were readily optimized and predesigned (embedded) inside the wells of 96-well plates.

The PCR plate was loaded into thermocycler CFX96 (Biorad, USA) and PCR amplification was carried out for 40 cycles involving denaturation step at 95 °C for 5 s, annealing and as well as extension at 60 °C for 30 s. Melting curve analysis was carried out at 60–95 °C (0.5 °C increments) for 5 s after 40 cycles completed. The reactions were performed in triplicate in 96-well plates. Fluorescence signal obtained was correlates with the template amount amplified. The cycle number at the threshold level of log-based fluorescence is defined as Cq number. This Cq number is used as data for further analysis. The Cq data gained from the PCR amplification were analysed using CFX Manager Software (Biorad, USA) to obtain the expression level of genes amplified. Relative normalized gene expression was quantified using Livak calculation method [25].migration, invasion and apoptosimigration, invasion and apoptosimigration, invasion and apoptosimigration, invasion and apoptosi

### Quantification of phosphoprotein expression via multiplex assay

Protein quantification was performed using Bio-Plex Pro Cell Signalling Assay Kit (Biorad, USA). The assay utilizes magnetic bead-based immunoassay for the detection of phosphoprotein and total protein in cells and tissue lysates.

The lysate of the samples was thawed and kept on ice. Tissue lysate control was reconstituted with 250 μL of deionized water, vortexed and incubated at room temperature for 20 min. All samples and lysate controls were centrifuged at 15000x *g* for 10 min at 4 °C before dispensing into the wells. Stock bead solutions (20x) were diluted to 1x by pipetting the required volume into the tube containing wash buffer (5472 μL) and vortexed for 15 s at medium speed. Afterwards, 50 μL of 1x beads solution were transferred into each well of the assay plate and washed 2 times with 200 μL wash buffer.

Sample lysate, lysate control and blank (antibody diluent) were gathered. Approximately, 50 μL of each test samples, control or blank were pipetted into each well accordingly. The plate was covered and incubated in the dark overnight (15–18 h) at room temperature. The plate was concurrently shaken at 900–1000 *rpm* for 30 s with reduction speed to 300–450 *rpm* during the incubation time. On the next day, the Bio-Plex system was warmed up and calibrated as described in the manual. Meanwhile, all reagents and diluents were kept at room temperature. Each detected antibody 150 μL (20x) was diluted into 2850 μL antibody diluent (1x) and vortexed. The plate was washed three times with 200 μL wash buffer and 25 μL diluted targeted antibody was added into each well of the assay plate accordingly. The plate was covered with sealing tape and incubated in the dark for 30 min at room temperature. The plate was concurrently shaken at 900–1000 *rpm* for 30 s with reduced speed to 300–450 *rpm* during the incubation time. While incubating the antibodies, Streptavidin-PE (SA-PE) 100x (60 μL) was diluted with 5940 μL antibody diluent (1x) and vortexed.

Afterwards, the sealing tape was removed, and the plate was washed three times with 200 μL wash buffer. 50 μL of SA-PE (1x) was added into each well of the assay plate. The plate was then covered with sealing tape and incubated in the dark for 10 min at room temperature. The plate was concurrently shaken at 900–1000 *rpm* for 30 s with reduced speed to 300–450 *rpm* during the incubation time. After completing the incubation period, the plate was washed again three times with 200 μL wash buffer and 125 μL resuspension buffer was added into each well to resuspend beads for plate reading. The plate was then covered with new sealing tape and shake at 900–1100 rpm for 30 s. After shaking, the sealing tape was removed and the plate was inserted into Bio-Plex 200 (Biorad, USA) for absorbance reading. Results obtained were recorded as median fluorescence intensity (MFI) emitted during the excitation of the bead. Data acquisition and analyses were conducted and interpreted by using Bio-Plex Manager software (Biorad, USA).

The complete procedure of the assay was conducted according to the protocol provided in the instruction manual inside the kits and can be accessed for details at http://www.bio-rad.com/webroot/web/pdf/lsr/global/english/primePCR/LIT10026370.pdf, http://www.bio-rad.com/webroot/web/pdf/lsr/literature/10024929.pdf and http://www.bio-rad.com/webroot/web/pdf/lsr/literature/Bulletin_6285.pdf. All kits had been optimized for high sensitivity and specificity.

### Statistical analysis

Statistical analysis was performed using SPSS version 21.0 for Windows (IBM, USA). Data were expressed as mean ± standard error of mean (S.E.M). The statistical difference of tumor incidence in in vivo data was evaluated by Chi square test. Whereas, the mean difference of tumor volume, tumor regression and tumor burden as well as molecular expression data between groups were analyzed with one-way ANOVA followed by post hoc test using Least Significant Difference (LSD). Fold-change ratio obtained from gene expression data was considered significantly upregulated and downregulated when the value is more than 2.0 and less than 0.5 fold respectively [26]. All data were considered statistically significant at *p* < 0.05.

## Results

### Antitumor-promoting effect of annonacin on DMBA/TPA induced mice tumorigenesis

Generally, there was no remarkable difference in the body, liver and kidney weights of mice in Group I, II, III and V throughout the experimental period of 22 weeks. However, there was a significant increase in the bodyweight (*p* < 0.05) of animals treated in Group IV (treatment control) when compared to Group I (vehicle control) (Table 1). In Group II (carcinogen control), the ratio of organ to final body weight i.e. liver was shown to be significantly elevated to 0.07 (*p* < 0.05) when compared to Group I (vehicle control) as shown in Table 1. The elevation of body and organ weights in both Group III (annonacin, 85 nM) and Group V (reference, curcumin 10 mg/kg [equivalent with 6.2 mM]) was not significant when compared to Group I (vehicle control).

**Table 1:** Effect of annonacin on the promotion stage of DMBA/TPA-induced mouse skin tumorigenesis after 22 weeks of treatment

Parameter/ Groups	No. of animals		Body weight (g)		Ratio to final body weight		Tumor latency (week)	Tumor incidence (%)	Tumor burden	Tumor volume (mm^3^)	Tumor regression
	Initial	Effective	Initial	Final	Liver	Kidney					
I (vehicle control)	10	10	23.8 ± 0.6^a^	31.7 ± 1.3^a^	0.05^a^	0.01^a^	–	–	–	–	–
II (carcinogen control)	10	10	24.0 ± 0.6^a^	32.4 ± 0.7^ab^	0.07^b^	0.02^a^	7^a^	100^a^	2.2 ± 0.4^a^	7.9 ± 1.7^a^	0.61 ± 0.3^a^
III (treatment, annonacin)	10	10	22.6 ± 0.5^a^	29.6 ± 1.2^a^	0.05^a^	0.01^a^	9^b^	50^b^	1.6 ± 0.3^a^	1.7 ± 0.4^b^	0.96 ± 0.4^a^
IV (treatment control)	10	10	23.6 ± 0.6^a^	35.3 ± 1.0^b^	0.05^a^	0.01^a^	–	–	–	–	–
V (reference, curcumin)	10	10	23.4 ± 0.7^a^	31.7 ± 1.4^a^	0.05^a^	0.01^a^	8^ab^	70^ab^	1.8 ± 0.3^a^	1.9 ± 0.4^b^	1.3 ± 0.4^a^

Values expressed as mean ± S.E.M (*n* = 10). Means between groups that have no superscript in common are significantly different (Tukey’s LSD, *p* < 0.05)

In terms of tumor latency, the first tumor was observed at 7 weeks of experimental period in Group II (carcinogen control). Group III (annonacin) and group V (reference, curcumin) significantly delayed the onset of tumor formation by 2 weeks and 1 week in comparison to Group II (carcinogen control) respectively, as shown in Table 1. Prior to euthanization, the tumor incidence, tumor burden, tumor volume and tumor regression were measured and calculated. Group II (carcinogen control) exhibited 100% of tumor incidence with mean tumor burden (2.2 ± 0.4) and tumor volume (7.9 ± 1.7 mm^3^) observed at the end of experimental period of 22 weeks. Interestingly, Group III (annonacin) was able to significantly reduce the tumor incidence to 50% and tumor volume to 1.7 ± 0.4 mm^3^, yet insignificantly reduced the tumor burden (1.6 ± 0.3) when compared to Group II (carcinogen control). Meanwhile, in Group V, topical application with the reference compound (curcumin) was able to slightly reduce tumor incidence to 30% and mean tumor burden to 1.8 ± 0.3. Nevertheless, volume of the tumors was small for the Group V as compared to the Group II (carcinogen control). In addition, there was no incidence of tumor development observed throughout the experimental period in both Group I (vehicle control) and Group IV (treatment control). Data for the morphological analysis is depicted in Table 1.

In comparison with Group V (reference, curcumin), Group III (annonacin) exhibited no significant difference in tumor incidence, tumor burden and tumor volume, respectively which may indicate the resemblance of both phytocompounds in terms of their antitumor promoting effects. Furthermore, there was no significant tumor regression observed between Group III (annonacin) and Group V (reference, curcumin), when compared to Group II (carcinogen control). However, curcumin exhibited greater regression activity in comparison to annonacin (Table 1).

### Effect of annonacin on histopathological assessment of skin tissue

**Fig. 1:**
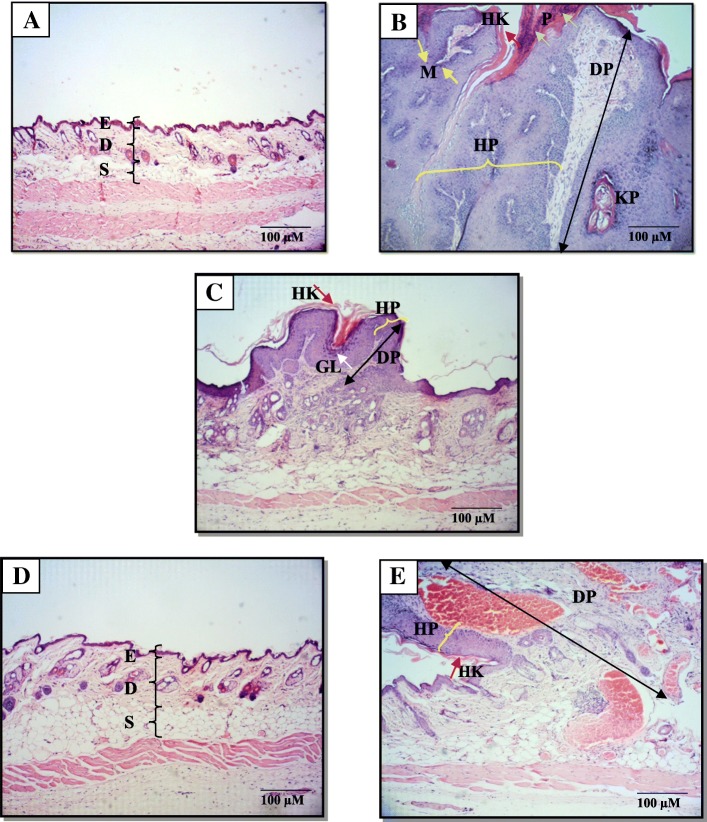
Micrographs of H&E skin tissues sections treated with (**a**) acetone (Group I; vehicle control) showed normal skin thickness. (**b**) DMBA/TPA (Group II; carcinogen control) showed huge hyperplastic epidermal layer, with increased number of keratinocyte (hyperkeratosis) and parakeratosis, increased size with huge extension of the dermal papillae, presence of keratin pearls and mark mitotic activity. (**c**) 85 nM annonacin (Group III; treatment) showed mild hyperplastic epidermal layer, mild dermal papillae extension, mild hyperkeratosis and prominent granular layer. (**d**) 85 nM annonacin without DMBA/TPA induction (Group IV; treatment control) showed the closest resemblance of skin thickness and morphology to Group 1. (**e**) 10 mg/kg curcumin (Group V; reference) showed moderate hyperplastic epidermal layer with increased number of keratinocyte, increased size with huge extension of dermal papillae. Note: [hyperplastic epidermal layer (HP) marked with yellow curly bracket], [hyperkeratosis (HK) marked with red arrow], [parakeratosis (P) marked with green arrow], [dermal papillae (DP) marked with black double arrow, keratin pearl (KP), [mitotic activity (M) marked with yellow arrow], [granular layer (GL) marked with white arrow], epidermis (**e**), dermis (**d**) and subcutaneous (S)

Histopathological evaluation on the skin tissues of control and treated mice in each group was further performed to confirm the results from the morphological observation, as depicted in Fig. 1a-e. Skin tissues in Group I (vehicle control) and Group IV (treatment control) resembled normal skin thickness, well defined subcutaneous tissue as well as an intact epidermal layer with no presence of hyperplasia, hyperkeratosis and parakeratosis (Fig. 1a and d).

Whilst, the tumor loaded skin tissues in mice of Group II, III and V were histologically identified as squamous papilloma known as benign tumor of the skin. This feature was observed during the first tumor onset until 22 weeks of experimentation period. However, severe hyperplasia with prominent hyperkeratosis, parakeratosis and presence of big keratin pearls formation was observed in Group II (carcinogen control). There was also a prominent extension of dermal papillae, increased in size of the cell, and increased of papillary dermis with a marked increase of mitotic activity at the adjacent border of the dermal layer seen in Group II (Fig. 1b). In Group III (annonacin), mild hyperplasia was also noted with mild hyperkeratosis and a prominent granular layer was also seen in this group with no keratin pearls and mitotic activity present (Fig. 1c). However, the skin tissues from Group V (reference, curcumin), exhibited only moderate epidermal hyperplasia with increased of papillary dermis and few extension of dermal papillae without any prominent hyperkeratosis, parakeratosis, keratin pearl and mitotic activity observed as shown in Fig. 1e. The size of papilloma in Group III (annonacin) was relatively small when compared to Group II and Group V.

### Effect of annonacin treatment on DBMA/TPA-induced modulations on gene expression

**Fig. 2:**
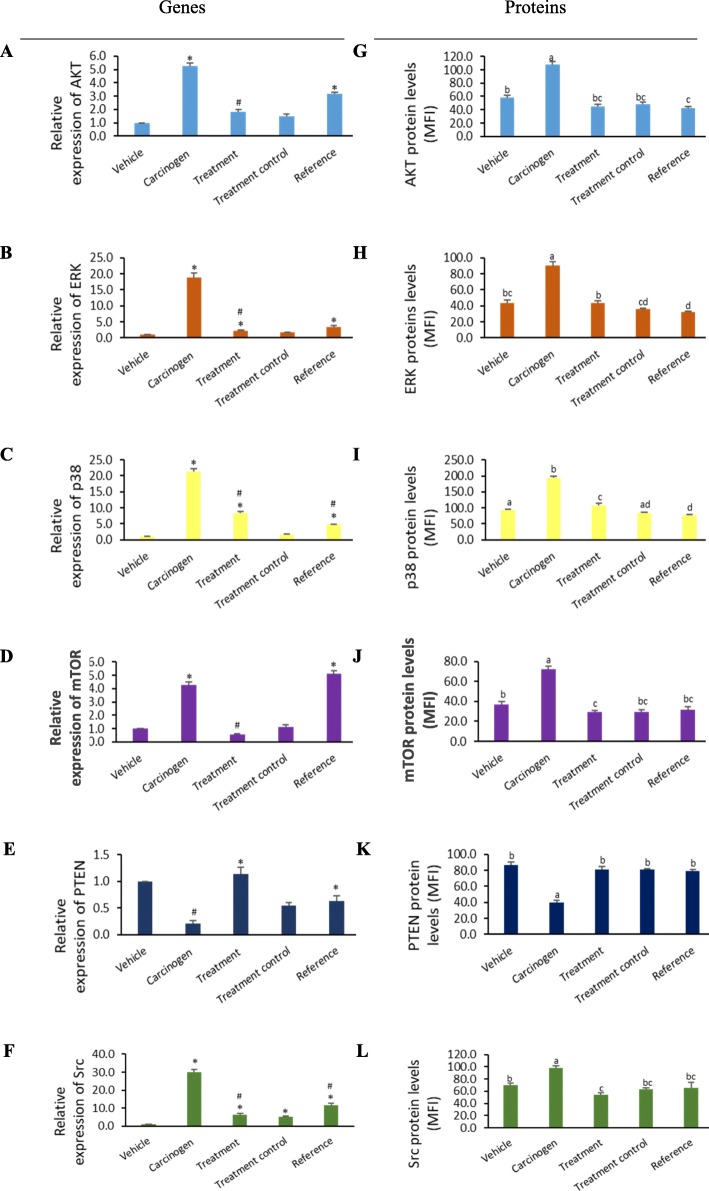
Annonacin showed significant modulation of molecular targets involved in mouse skin tumorigenesis. qRT-PCR and Bio-Plex MAGPIX revealed down-regulation of AKT (**a**, **g**), ERK (**b**, **h**), p38 (**c**, **i**), MTOR (**d**, **j**), Src (**f**, **l**) and up-regulation of PTEN (**e**, **k**) genes and protein respectively in annonacin treated mouse skin tumorigenesis. * (significant up-regulation - values expressed as relative individual gene expressions to vehicle control group). # (significant down-regulation- values expresses as relative individual gene expression to carcinogen control group). Values with different superscripts letter among the groups are statistically significant (*p* < 0.05). All experiments were performed in triplicate

Gene expression analysis via qRT-PCR showed that the application of TPA on mice skin initiated with DMBA in Group II (carcinogen control) resulted in significant upregulation (> 2-fold) of *AKT, ERK, mTOR, p38*, and *Src*, as well as downregulation (< 0.5-fold) of *PTEN* when compared to Group I (vehicle control) as shown in Fig. 2a-f.

Interestingly, topical application of 85 nM annonacin in Group III, prior to each TPA application, significantly downregulated the expression of *AKT, ERK, mTOR* and *p38* to 0.3-, 0.1-, 0.1- and 0.4-fold, respectively, when compared to Group II (carcinogen control) (Fig. 2a-d). Group III (annonacin) showed better downregulation of *AKT, ERK* and *mTOR* than Group V (reference, curcumin) indicating its higher efficacy in modulating and restoring the aforementioned target genes to the normal level.

In contrast, a slight increase of *AKT, ERK, p38*, and *mTOR* expressions was observed in Group IV (treatment control), but the increment of the expressions was not significant in comparison to Group I (vehicle control). Annonacin treatment in mice of Group III was also shown to significantly upregulate *PTEN* expressions to 7-fold when compared to Group II (carcinogen control), signifies the capability of annonacin to restore *PTEN* activity almost to the normal level.

Treatment of annonacin also significantly downregulated *Src* expression by 0.2-fold when compared to Group II (carcinogen control). Moreover, *Src* gene expressions in Groups III, IV and V were significantly downregulated when compared to Group II (carcinogen control) as shown in Fig. 2f. Annonacin also showed better downregulation of *Src* expression than curcumin. Contrarily, in Group III, IV and V, increase of *Src* expressions were observed when compared to Group I (vehicle control), however the expression is still lower than Group II (carcinogen control).

### Effect of annonacin treatments on DBMA/TPA-induced modulations on protein expression

The gene expression of amplified targets via qRT-PCR was further confirmed with phosphoprotein expression level via multiplex protein assay. Consistent with the results obtained from the gene expression analysis, AKT, ERK, mTOR, p38 and Src protein levels also showed similar trend of significant elevation whilst simultaneously suppressed PTEN in Group II (carcinogen control) when compared to Group I (vehicle control), confirming these targets were involved in tumorigenesis in DMBA/TPA induced mice at both gene and protein levels.

Remarkably, annonacin treatment on DMBA/TPA-induced mice in Group III was able to significantly suppress more than 50% of AKT, ERK and mTOR, Src and p38 protein levels (Fig. 2g-l), and also significantly augmented more than 100% of PTEN protein level respectively when compared to Group II (carcinogen control). In comparison to Group V (reference, curcumin), there was no significant changes of aforementioned protein expressions observed between both groups (except ERK and p38), suggesting annonacin’s comparable effect to curcumin in the modulation of AKT, mTOR, PTEN and Src proteins expression. Group IV (treatment control) did not exhibit any significant changes in all proteins as compared to Group I (vehicle control), indicating its non-influential effect in triggering any major proteins responsible in carcinogenesis when individually applied on normal healthy mice.

### Histopathological effect of annonacin in liver and kidney

In general, significant inflammation was observed in mice of all groups in both the liver and kidney. Inflammation is characterized by the presence of neutrophils and lymphocyte in the inflamed area of the tissues. The highest mean scores of 1.8 ± 0.1 and 2.3 ± 0.2 were obtained in liver (Table 2) and kidney (Table 3), respectively, in Group II (carcinogen control). In contrast, there was no significant difference of inflammation observed in Group III, IV and V in both organs.

**Table 2:** The distribution and mean scores of histopathological changes due to toxicity in mice liver of each group

Features/ Groups	Inflammation		Degeneration		Necrosis		Sinusoidal dilatation		Microvesicular steatosis	
	Distribution (*n* = 10)	Mean score ± S.E.M	Distribution (*n* = 10)	Mean score ± S.E.M	Distribution (*n* = 10)	Mean score ± S.E.M	Distribution (*n* = 10)	Mean score ± S.E.M	Distribution (*n* = 10)	Mean score ± S.E.M
I (vehicle control)	10	1.0 ± 0.0	0	0.0 ± 0.0	0	0.0 ± 0.0^a^	0	0.0 ± 0.0	0	0.0 ± 0.0^b^
II (carcinogen control)	10	1.8 ± 0.1^a^	2	0.3 ± 0.2^a^	0	0.0 ± 0.0^a^	8	1.2 ± 0.8^a^	10	1.7 ± 0.2^a^
III (treatment, annonacin)	10	1.0 ± 0.0	0	0.0 ± 0.0	0	0.0 ± 0.0^a^	0	0.0 ± 0.0	4	0.4 ± 0.2^bc^
IV (treatment control)	10	1.0 ± 0.0	0	0.0 ± 0.0	0	0.0 ± 0.0^a^	0	0.0 ± 0.0	2	0.2 ± 0.1^b^
V (reference, curcumin)	10	1.2 ± 0.5	0	0.0 ± 0.0	0	0.0 ± 0.0^a^	0	0.0 ± 0.0	4	0.4 ± 0.2^bc^

Values expressed as mean ± S.E.M (*n* = 10). Means between groups that have no superscript in common are significantly different (Tukey’s LSD, *p* < 0.05)

**Table 3:** The distribution and mean scores of histopathological changes due to toxicity in mice kidney of each group

Features/ Groups	Inflammation		Degeneration		Necrosis		Bowman capsule dilatation		Glomeruli cellularity	
	Distribution (*n* = 10)	Mean score ± S.E.M	Distribution (*n* = 10)	Mean score ± S.E.M	Distribution (*n* = 10)	Mean score ± S.E.M	Distribution (*n* = 10)	Mean score ± S.E.M	Distribution (*n* = 10)	Mean score ± S.E.M
I (vehicle control)	8	0.8 ± 0.1	0	0.0 ± 0.0	0	0.0 ± 0.0^a^	0	0.0 ± 0.0	0	0.0 ± 0.0
II (carcinogen control)	10	2.3 ± 0.2^a^	2	0.2 ± 0.1^a^	0	0.0 ± 0.0^a^	6	0.6 ± 0.2^a^	8	1.0 ± 0.2^a^
III (treatment, annonacin)	9	0.9 ± 0.1	0	0.0 ± 0.0	0	0.0 ± 0.0^a^	0	0.0 ± 0.0	0	0.0 ± 0.0
IV (treatment control)	6	0.5 ± 0.2	0	0.0 ± 0.0	0	0.0 ± 0.0^a^	0	0.0 ± 0.0	0	0.0 ± 0.0
V (reference, curcumin)	10	1.2 ± 0.1	0	0.0 ± 0.0	0	0.0 ± 0.0^a^	0	0.0 ± 0.0	0	0.0 ± 0.0

Values expressed as mean ± S.E.M (*n* = 10). Means between groups that have no superscript in common are significantly different (Tukey’s LSD, *p* < 0.05)

**Fig. 3:**
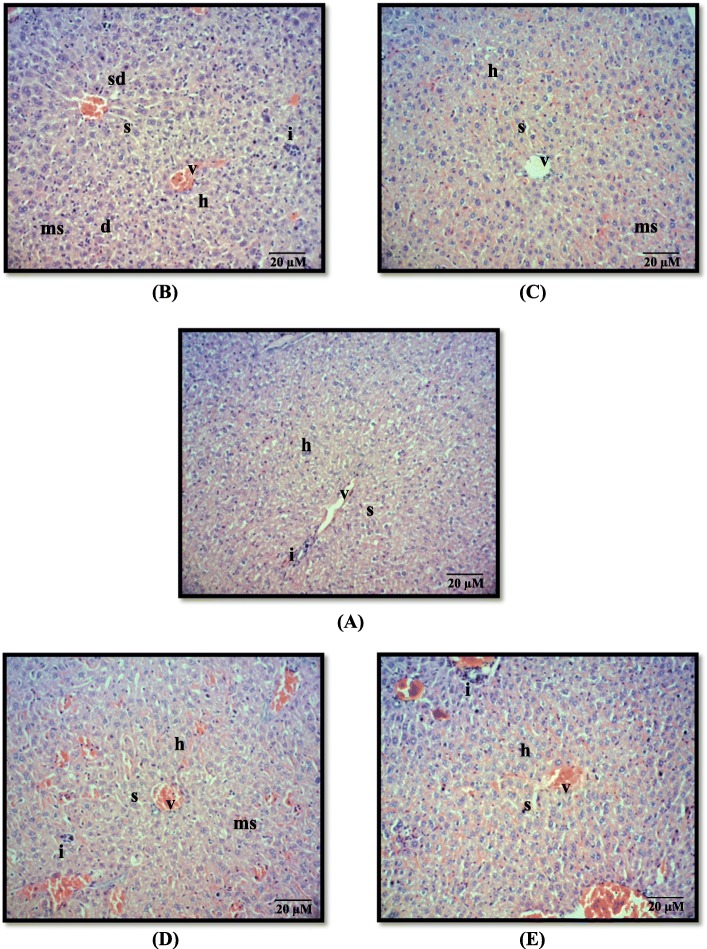
Micrograph of H&E sections from mice liver treated with (**a**) acetone (Group I, vehicle control); (**b**) DMBA/TPA (Group II, carcinogen control); (**c**) 85 nM annonacin (Group III, treatment); (**d**) 85 nM annonacin only (Group IV, treatment control); (**e**) 10 mg/kg curcumin (Group V, reference). Note: inflammation (i), degeneration (d), sinusoidal dilatation (sd), microvesicular steatosis (ms), central vein (v), hepatocyte (h) and sinusoid (s). Magnification × 100

**Fig. 4:**
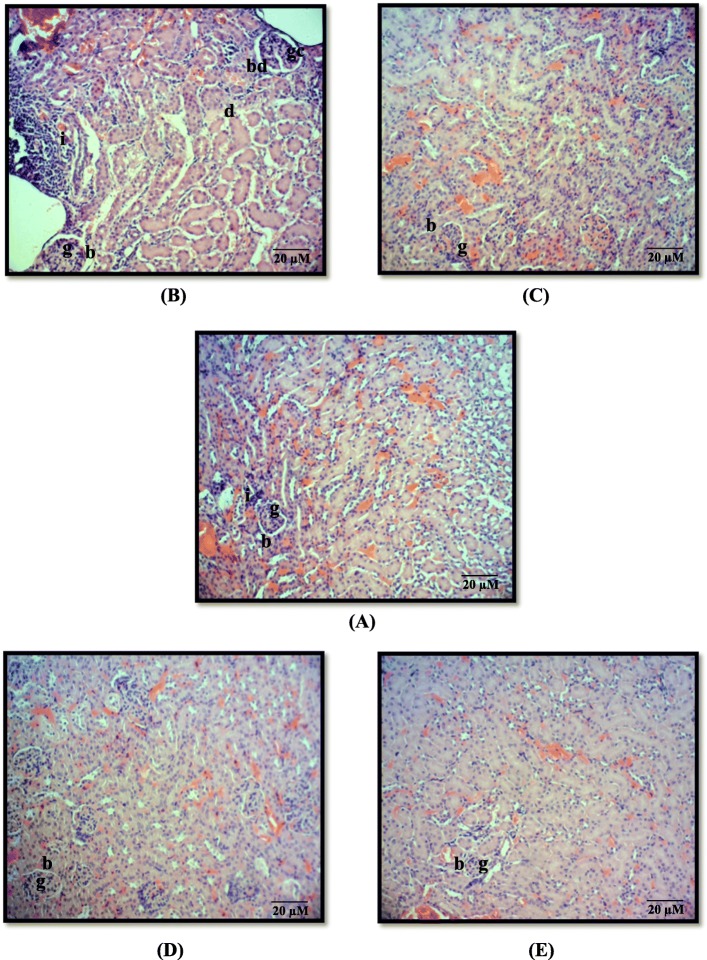
Micrograph of H&E sections from mice kidney treated with (**a**) acetone (Group I, vehicle control); (**b**) DMBA/TPA (Group II, carcinogen control); (**c**) 85 nM annonacin (Group III, treatment); (**d**) 85 nM annonacin only (Group IV, treatment control); (**e**) 10 mg/kg curcumin (Group V, reference). Note: inflammation (i), degeneration (d), Bowman‘s capsule dilatation (bd), glomerular cellularity (gc), glomerulus (g) and Bowman’s capsule (b). Magnification × 100

No cell degeneration was found in both liver and kidney in mice of each group except in Group II (carcinogen control) with mean scores of 0.3 ± 0.2 and 0.2 ± 0.1 respectively. Cell degeneration is identified as a swollen cell with a displaced nucleus. On top of that, there were no necrotic features observed in both liver and kidney of the mice in all groups in which necrotic cells can be ubiquitously characterized by the pyknotic nucleus, membrane disruption and cytoplasmic coagulation (Fig. 3 and Fig. 4).

Group II showed significant sinusoidal dilatation (widening of capillaries around the sinusoid) as shown in Fig. 3b with a mean score of 1.2 ± 0.8 in the liver. Group II also showed significant Bowman’s capsule dilatation in the kidney as shown in Fig. 4b with a mean score of 0.6 ± 0.2. Moreover, there were also significant hepatocytes with microvesicular steatosis (cell cytoplasm become vacuolated because of fat accumulation) and increased glomerular cellularity of nephron observed in Group II (Figs. 3b and 4b).

The toxicity effect of annonacin treatment alone to the hepatocytes and nephron (Group IV) was also observed. Annonacin was shown to exhibit insignificant very mild microvesicular steatosis with score of 0.2 ± 0.1, indicating its non-hazardous effects in mice hepatocyte. Topical application of annonacin for 22 weeks in Group IV, was not able to cause liver and kidney toxicities, evidenced by the results of histopathological analysis. Besides, kidneys dissected from Group III and Group V also showed no evidence of Bowman’s capsule dilatation and increased glomerular cellularity (Fig. 4c and e). The details of histopathological changes of both liver and kidney in each group are shown in Table 2 and Table 3, respectively.

## Discussion

In this study, results showed that topical application of tumor initiator, DMBA (390 nM) followed by the promoter, TPA (1.7 nM) applied on mice, twice a week for 22 weeks produced multiple skin papillomas. Mouse skin tumorigenesis model is the best recognized in vivo models for the study of the chronological and stepwise development of tumors from initiation to progression stage [13]. To date, this model is still being a model of choice to study the antitumor promoting effect of either synthetic or plant-based antineoplastic agents [14–16]. This due to the capability of his model mimics the features of multi-stage carcinogenesis in humans as humans are typically exposed to various doses of both carcinogens and promoting agents [17, 24]. This model also enables tumor development and progression to be monitored visually throughout the life span of the mouse.

TPA induces the production of superoxide free radicals that have been implicated as the causative factor in various diseases including skin cancer [27]. In comparison to the initiation stage which is an irreversible process, the promotion stage occurs over to an extended period of time that may be reversible during the tumorigenesis process [28]. Upon induction by the tumor promoter, several key events have been recognized in skin tumor promotion including epidermal hyperplasia, keratinocyte proliferation, inflammation and oxidative stress [19].

In the current study, the application of annonacin 85 nM was significantly reduced the tumor incidence and tumor volume in DMBA/TPA-induced tumor on mice skin. This finding was in agreement with previous findings where annonaceous acetogenins including annonacin have been reported to be responsible for the potent anticancer activities in many cancer cell lines through the inhibition of mitochondrial complex I enzyme, to cause energy depletion [7, 8]. The dose of 85 nM used was in accordance with many previous established reports that employing similarly in vivo model using bioactive compound in the antitumor promoting study [29–31].

In another study by Wang et al. [32], annonacin (10 mg/kg) was able to inhibit mouse lung cancer by 57.9% in an in vivo model using hybrid mice (BDF-1) when administered orally. In addition, several studies on the antitumoral effect of *Annona muricata* leaves extract (AML) were conducted in various in vivo model [33–35]. Recently, the antitumoral effect of *Annona muricata* was shown to be primarily due to the activity of annonacin [35].

Histopathological analysis revealed that the appearance of the tumor in each Group II, III and V were morphologically identified as papilloma, which is characterized as a small benign solid, with a clear-cut border growing exophytically from the epidermal layer of the skin. Representative microphotographs of H&E sections from each group were depicted in Fig. 1. Notably, the current regimen in our study did not lead to the formation of SCC. This may be possibly due to the low DMBA/TPA dosage applied, shorter period of observation and the type of mouse strain used in this experiment, which are accounted as crucial factors for the development of SCC [13].

Sustained cell proliferation is one of the major characteristics in skin tumor promotion [13, 19]. The occurrence of this event is a response to the repetitive application of tumor promoter, preceded by inflammation and followed by skin papillomatosis that eventually leads to malignant transformation (SCC) [19]. As shown in Fig. 1b, severe increased keratinocyte proliferation depicted via epidermal thickening was noted in Group II (carcinogen control), in which this event is caused by the upregulation of AKT, ERK, p38, mTOR and Src [18, 19]. Interestingly, treatments with 85 nM annonacin (Fig. 1c) and 10 mg/kg curcumin (equivalent with 6.2 mM) have significantly reduced keratinocyte proliferation prior to the induction with DMBA/TPA for 22 weeks, respectively (Fig. 1d).

During skin tumorigenesis, single and multiple applications of tumor promoter leads to the activation of the Ras/Raf/MAPK/ERK and PI3K/AKT/mTOR pathways [19, 37] In this study, once EGFR is activated by the tumor initiator (DMBA) it will phosphorylate the upper stream targets in PI3K/AKT/mTOR and Ras/Raf/MAPK pathways which in majorly consisted of kinases protein. Protein kinases are responsible for the mechanism of phosphorylation. They are activated by phosphorylation which in turn activates a cascade of events leading to the phosphorylation of downstream effectors including regulatory protein than controlling gene expression [38].

Phosphorylation is one of the most important processes in post - translational modification of protein. Additional of phosphate group into the protein structure will eventually change the protein conformation and thus alter its activity when interacting with other molecules. This process plays a crucial role in many cellular processes including protein activity, subcellular localization and transmits signal downstream to the reaction path. Phosphorylation may act in two different ways, either activates or inactivate the protein [38].

Phosphorylation is a major event in activation of PI3K/AKT/mTOR and Ras/Raf/MAPK pathways. Both pathways play an important role in cell survival, cell proliferation, apoptosis and protein synthesis through AKT activation that phosphorylates their downstream proteins Bad, Bax, Caspase and mTOR. Cell proliferation and inflammation are also activated through RAS activation through cascade phosphorylation of Raf, MEK, ERK, p38 and NFκB (Additional file 1: Fig. S1) [19, 37]. Therefore, the capability of annonacin to inhibit the activation of AKT, ERK, mTOR and p38 will halt the cascade phosphorylation of other downstream proteins that involve in PI3K/AKT/mTOR and Ras/Raf/MAPK pathways.

Besides, according to Ko et al. [10], the molecular mechanism of annonacin in the regulation of gene protein expressions can be studied through binding assay and transcriptional activation assays. Downregulation of ERK, JNK and STAT3 protein expression by annonacin in the study [10] is due to the regulation of its transcriptional activity trough phosphorylation inhibition. Similar result was also reported by Chung et al. [38], where they found that reduced phosphorylation of ERK protein in endometrial cancer cell lines (HEC-1A, EC6-ept, and EC14-ept) treated with annonacin resulted with low expression of ERK.

It is ubiquitously known, overexpression of ERK, AKT, mTOR, p38 and proto-oncogene (Src) as well as the loss of tumor suppressor function (PTEN) proteins have been implicated in wide number of cancers in human including skin cancer [36]. In addition, curcumin has been reported to exert remarkable effects in modulating various protein kinases and antiapoptotic proteins that are crucial for cell growth such as AKT, ERK, p38, mTOR and Src either through inhibition or activation of phosphorylation [39–42]. As curcumin has been known effectively to hit several deviant targets simultaneously in many cancer pathways and models, therefore, annonacin, is suggested to exert the similar mechanism like curcumin in regulating gene and protein expressions.

In addition, the downregulation and upregulation of protein in this study could be explained through ATP usage during phosphorylation. Annonacin also known of its anticancer property as an inhibitor of complex I enzyme, to cause energy depletion [7]. The phosphorylation process itself require phosphate group that derive from the hydrolysis of ATP due to enzymatic activity of kinase [38]. Since annonacin causes ATP depletion in tumor cells, thus phosphorylation will be interfered and eventually will change the protein activity and signal transduction process. This was explained by the study done by Liu et al. [43]. The study has shown that, polyether mimicking acetogenin (AA005) decrease the phosphorylation of mTOR through suppression of ATP production in colon cancer cells lines [37].

It is also widely known that, there is a strong association between inflammation and tumorigenesis/carcinogenesis, where NF-κB, a family of transcription factors plays an essential role in inflammation as well as in immunity [44], which was later identified to be responsible for the various steps in cancer initiation and progression [19]. *Annona muricata* has been known to act effectively as an anti-inflammatory [45, 46], where inflammation has also been known to be implicated in tumorigenesis through modulation of NF-κB signaling pathways that commonly associates with ERK, p38 MAPK, Ras, and PI3K/AKT pathways [47]. Moreover, *Annona muricata* was reported to inhibit the inflammatory process through inhibition of both COX-1 and COX-2 [45]. Consistently, the complement cascade activation of COX-2 by NF-κB has been shown to activate PGE_2_ in which, it has been shown to play a role in inducing keratinocyte proliferation through crosstalk via multiple upper stream signaling pathways including PI3K/AKT, ERK and EFGR [19, 48].

Likewise, the downregulation of p38, AKT and ERK expression by annonacin in this study, is also suggested to affect COX-2 suppression. Moreover, ERK and p38 have been shown to modulate NF-κB and COX-2 activation [48, 49].

On the other hand, COX-2 activity can also be modulated under the influence of Src activity [50]. Src can activate most of the crucial cascades such as PI3K/AKT as well as Ras/Raf/MAPK that function in cell proliferation, cell cycle progression and survival [50, 51]. Matsumoto et al. [22], also reported that, activation of Src is a crucial event in epidermal hyperplasia in skin tumor promotion. Moreover, reduced activity of Src in this study could be explained by inhibition of phosphorylation caused by insufficiency of ATP as annonacin inhibits the production of ATP in the mitochondria [7, 8]. Therefore, annonacin is suggested to undergo the similar mechanism in preventing the activation of ERK and AKT pathways through inhibition of Src activation. This event eventually inhibits keratinocyte proliferation, which is a crucial process during the promotion stage in tumorigenesis.

A study has demonstrated the restoration of PTEN and simultaneous inhibition of P13K expression resulted in the antitumor effect of selected natural compounds [52]. In addition, PTEN has been identified to regulate many cellular functions including growth, adhesion, migration, invasion and apoptosis [53]. The role of PTEN in apoptosis is well perceived. Restoration of PTEN expression has also been described to downregulate PI3K signaling, thus causing tumor cell death and cell cycle arrest at the G1 stage due to the cleavage of PIP3 [54]. Modulation of prominent tumor suppressor gene, *PTEN* by annonacin, has been suggested to be linked with AKT expression as evidence in this study (Fig. 2), where the level of *PTEN* expression was inversely correlated with AKT *phosphorylation. This trend was in accordance with previous studies that shown* AKT Inhibitor VIII blocked phosphorylation of AKT and Bad, but not PTEN [55]. This could explain the increased expression pattern of compared to AKT in Group III (Fig. 2).

In terms of toxicity, organ weight and/or with ratio to body weight has been widely accepted for evaluation of test substance-associated toxicities [56, 57]. Increased in body weight due to toxicity has been linked with the increased weight of several vital organs including liver, kidney, heart, brain and spleen [56]. This study shown that, non-induced mice treated with annonacin alone (Group IV) showed significant changes in body weight gain compared to vehicle control mice (Group I). However, no significant changes in the liver and kidney weights were observed in both groups as mentioned earlier, indicating the non-influential effect of weight gain with either toxicity or adverse effect. This generalization is supported by the studies conducted by Chan [58] and Hoffman [59], who explained the possible toxicity is due to the significant changes in weights of both liver and kidney. Meanwhile, our current results found that annonacin failed to show any hyperplasic and cytomegalic changes in both organs when examined grossly and microscopically. Both features are known to cause body weight gain due to toxicity [58, 59]. Interestingly, no other trace of hepatocyte toxicity features, i.e. irreversible injury (necrosis) was seen in both liver and kidney in all groups. No degeneration and sinusoidal dilatation were found in the liver of mice treated with annonacin (Group IV, treatment control) indicating its non-toxicity when topically applied for 22 weeks.

Current results also showed a very mild score of inflammation and microvesicular steatosis observed in the liver (Group IV) as compared to the carcinogen control (Group II). The weak occurrences of inflammation scattered in few areas of both liver and kidney tissues in all groups are considered normal condition [60, 61]. In addition, very mild occurrence of microvesicular steatosis in the liver observed in mice treated with annonacin alone (Group IV) is also deduced as a normal condition, mainly regarded as an adaptive mechanism towards the accumulation of toxic compound. Accumulation of toxic substance will lead to oxidative stress which eventually causes mitochondrial dysfunction along with altered oxidative phosphorylation and impairment of mitochondrial β oxidation [62]. Nevertheless, increased concentrations of intracellular fatty acids may be directly toxic to hepatocytes [63]. According to Kaplowitz [64], liver cell damage may also be due to precipitation of metabolites from the toxic compound. Therefore, this may explain, that annonacin showed no significant sign of toxicity manifested by analysis of body and liver weight and microscopic liver histopathology. The insignificant marked inflammation and microvesicular steatosis are considered as normal adaptation mechanisms of hepatocytes due to the exposure of xenobiotic. Our findings were also in line with the study done by Hansra et al. [65], where they found treatment using *Annona muricata* decoction (10–12 dry leaves in water) for 5 years to a breast cancer patient, managed to stabilize the disease with no side effects observed.

Moreover, induction of DMBA/TPA in carcinogen control (Group II) mice irreversibly affected the kidney’s toxicity, manifested by significant increase of glomerular cellularity, Bowman’s capsule dilation and mild number of tubular cell degeneration, respectively. Our findings were in agreement with the previous study, where croton oil (a crude form that contain TPA) has also been reported to cause glomerulonephritis, thus led to an increase in cellularity (hypercellularity) and inflammation [66]. Glomerular hypercellularity is commonly marked with an increase of the number of endothelial, mesangial, and inflammatory cells [67]. Interestingly, there is no toxicity features such as Bowman‘s capsule dilatation, increase in glomerular cellularity and degeneration were observed in both Group III (annonacin) and IV (treatment control) as shown in Table 3. In addition, most of the histopathological observations of these tissues resemble the features characterized in Group I (vehicle control).

## Conclusions

Collectively, the current findings provide new insight of annonacin’s mechanism of action in suppressing tumorigenesis. Annonacin demonstrates down-regulation of AKT, ERK, mTOR, p38 and Src, whilst simultaneously prevents PTEN down-regulation at gene and protein levels. Modulation of these genes eventually promotes cell apoptosis in the skin tumor cells via inhibition of PI3K/AKT/mTOR signaling pathway in vivo. This is evidenced by delayed papilloma development, incidence as well as its reduction of volume. On the other hand, annonacin was found to be non-toxic to the liver and kidney and was able to protect skin from further development of neoplastic changes featured by the absence of mitotic activity, mild hyperplasia and mild hyperkeratosis. Finally, this study suggests that annonacin may serve as a potential therapeutic compound for the prevention and treatment of skin cancer.

## Additional files

Additional file 1: **Figure S1.** Depiction of molecular mechanism of antitumor promotion effect by annonacin in two-stage mouse skin tumorigenesis. (DOCX 72 kb)

## Data Availability

The datasheet generated or analysed during the current study are not publicly available due to prevention of third party misuse such as over claiming of natural based products by manufacturer and false article production by unethical researcher. Author is also planning to commercialize the research findings.

## Abbreviations

AKT: serine/threonine-protein kinase
AML: *Annona muricata* leaves extract
ANOVA: Analysis of variance
Bax: Bcl-2 Associated X
Bcl-2: B-cell lymphoma 2
BDF-1: Hybrid mice cross between female C57BL/6 and male DBA/2
cDNA: Complementary deoxyribonucleic acid
COX: Cyclooxygenase
Cq: Cycle number at the threshold level of log-based fluorescence
DMBA: 7,12-dimethylbenz[α]anthracene
DMSO: Dimethylsulfoxide
ER- α: Estrogen receptor *alpha*
ERK: Extracellular regulated kinases
GAPDH: Glyceraldehyde 3-phosphate dehydrogenase
GUSB: Glucuronidase beta
H & E: hematoxylin and eosin
HIF-1α: Hypoxia inducible factor 1 subunit alpha
HPLC: High Performance Liquid Chromatography
ICR: Institute of Cancer Research
LSD: Least Significant Difference
MAPK: Mitogen-activated protein kinases
MFI: Median fluorescence intensity
mTOR: Mechanistic target of rapamycin kinase
NF-κB: Nuclear factor kappa B
p38: P38 mitogen-activated protein kinases
PBS: Phosphate buffer saline
PCR: Polymerase chain reaction
PI3K: *Phosphoinositide* 3-kinases
PTEN: Phosphatase and tensin homologue
qRT-PCR: Quantitative real time- polymerase chain reaction
Raf: Rapidly accelerated fibrosarcoma
Ras: Rat sarcoma
S.E.M: Standard error of mean
SA-PE: Streptavidin, Phycoerythrin
SCC: Squamous cell carcinoma
SPSS: Statistical Package of Social Sciences
Src: Proto-oncogene tyrosine-protein kinase Src
TPA: *O*-tetradecanoylphorbol-13-acetate
