# Supplementary material for: Modulation of cancer signalling pathway(s) in two -stage mouse skin tumorigenesis by annonacin
Source: BMC Complement Altern Med. 2019 Sep 3;19:238. doi: 10.1186/s12906-019-2650-1 (PMC6724370; doi:10.1186/s12906-019-2650-1)
Supplement: Supplementary file 1 — Figure S1. Depiction of molecular mechanism of antitumor promotion effect by annonacin in two-stage mouse skin tumorigenesis. (DOCX 72 kb) [file 12906_2019_2650_MOESM1_ESM.docx]

**Animal Research: Reporting in Vivo Experiments**

| **No** | **Item** | **Remarks** |
| --- | --- | --- |
| 1 | Title | Modulation of cancer signalling pathway(s) in two -stage mouse skin tumorigenesis by annonacin  **(Page 1)** |
| 2 | Abstract | Background  Annonacin, an annonaceous acetogenin isolated from Annona muricata has been reported to be strongly cytotoxic against various cell lines, in vitro. Nevertheless, its effect against in vivo tumor promoting activity has not been reported yet. Therefore, this study was aimed to investigate antitumor-promoting activity of annonacin via in vivo two-stage mouse skin tumorigenesis model and its molecular pathways involved  Methods  Mice (n=10) were initiated with single dose of 7,12-dimethylbenz[α]anthracene (DMBA) (390 nmol/100 μL) followed by, a week later, repeated promotion (twice weekly; 20 weeks) with 12-O-tetradecanoylphorbol-13-acetate (TPA) (1.7 nmol/100 μl). Annonacin (85 nM) and curcumin (10 mg/kg; reference) were, respectively, applied topically to DMBA/TPA-induced mice 30 minutes before each TPA application for 22 weeks. Upon termination, Histopathological examination of skin, liver and kidney as well as genes and proteins expression analysis were conducted to elucidate the potential mechanism of annonacin  Results  With comparison to the carcinogen control, Annonacin significantly increased the tumor latency period and also reduced the tumor incidence, tumor burden and tumor volume, respectively. In addition, it also suppressed tumorigenesis manifested by significant reduction of hyperkeratosis, dermal papillae and number of keratin pearls on skin tissues. Annonacin also appeared to be non-toxic to liver and kidney. Significant modulation of both AKT, ERK, MTOR, p38, PTEN and Src genes and proteins were also observed in annonacin-targeted signaling pathway(s) against tumorigenesis.  Conclusions  Collectively, results of this study indicate that annonacin is a potential therapeutic compound targeting tumor promoting stage in skin tumorigenesis by modulating multiple gene and protein in cancer signaling pathways without apparent toxicity.  **(Abstract/Page 2/Line 1-27)** |
| **INTRODUCTION** | | |
| 3 | Background   1. Scientific background and rationale 2. Justification of model being used | Medicinal plants have been used since ancient age in folk medicine all over the world and there are many nations which are still practicing, utilizing and developing plant based treatment as well as the naturally sourced compounds for prophylactic and therapeutic purposes in various diseases including cancer. Chemotherapy plays an important role in the treatment of many cancer type including skin cancer. The chemical agents used in chemotherapy are selectively destructive to malignant cells, but these agents can also cause damage to the healthy normal cells, which results in adverse side effects that negatively impact compliance with cancer treatment as well as its well-being [1]. Therefore, there is an imperative need to find a strategy to resolve the problem. This has eventually led to the discovery of new plant-based chemopreventive agent as an ideal strategy, since plants are generally considered safe with low toxicity as supported by their regular usages since centuries ago. Anticancer compounds derived from terrestrial plants include polyphenols, brassinosteroids and taxol [2].  Two-stage skin cancer model consists of multistage tumorigenesis, offers an investigational framework to study the basic mechanism linked with the initiation, promotion and progression stages in animal. It is also beneficial in examining chemopreventive agents that potentially affect any stages of tumorigenesis with regard to understanding molecular mechanism and evolution of cancer cells, not only in skin cancer, but in other multistage human cancers such as prostate and colon cancer [3].  Number of evidence have demonstrated that *Annona muricata* L possesses pleiotropic effects such as anticancer, antiparasitic, pesticidal, antimicrobial and antiviral activities [4,50. Among these activities, anticancer activity has been intensively studied due to the exploration of cytotoxic compounds abundantly present in various parts of *Annona muricata* namely, annonaceous acetogenin.  Annonaceous acetogenins have been reported to exert cytotoxicity in various cancer cell lines such as lung, colon, pancreatic, prostate, and breast [4, 6]. For the past 30 years, several researchers have conducted numerous studies in elucidating the underlying mechanisms of actions of these compounds. Amongst the mechanisms reported are the inhibition of mitochondrial complex 1 enzyme, induction of apoptosis, blocking of the cell cycle through the halt at certain phases and inhibition of DNA topoisomerase [4].  Annonacin, a mono-tetrahydrofuran acetogenin is one of the major compound consists in *Annona muricata* leaf and seed and in other plants from Annonaceae family. It has been priorly reported to be actively cytotoxic against various cancer cell lines, *in vitro* [4, 6]. Despite its role as a potent inhibitor of the mitochondrial complex I in electron transport system, its other mechanisms in tumorigenesis have yet to be extensively explored. To the best of our knowledge, the mechanisms of annonacin in modulating tumorigenesis pathways include, upregulation of Bax and caspase-3 expression that lead to apoptosis [7]; inhibiting ER-α, cyclin D1 and Bcl-2 protein expressions [8]; inhibits HIF-1α and MTOR activation [9]; and downregulation of ERK protein expression [10], that eventually lead to cancer cell apoptosis.  To date, all of the mechanisms previously reported on annonaceous acetogenin including annonacin, were based on *in vitro* results. There is no study conducted in elucidating the mechanism of action of annonaceous acetogenin in any *in vivo* model yet. Thus, this current study was aimed to investigate the antitumor promoting effect and toxicity of annonacin, in two stage mouse skin tumorigenesis, as well as its proposed molecular pathways in suppressing/inhibiting the skin tumorigenesis.  Alteration to cancer proliferative signaling pathways, RAS/RAF/MAPK/ERK and PI3K/AKT/MTOR together mutation of tumor suppressor genes have been implicated in various human cancers including skin cancer [11,12]. These signaling pathways impart cancer cells with neoplastic progression through uncontrolled cell proliferation and inhibition of apoptosis [13]. Since RAS/RAF/MAPK/ERK and PI3K/AKT/MTOR signalling pathways are known to play a major role in cell proliferation and apoptosis, the effect of annonacin on selected targets of these pathways were further studied in both gene and protein levels.  **(Background/Page 3-5/Line 32-82)** |
|  |  | Two-stage skin cancer model consists of multistage tumorigenesis, offers an investigational framework to study the basic mechanism linked with the initiation, promotion and progression stages in animal. It is also beneficial in examining chemopreventive agents that potentially affect any stages of tumorigenesis with regard to understanding molecular mechanism and evolution of cancer cells, not only in skin cancer, but in other multistage human cancers such as prostate and colon cancer [3].  **(Background/Page 4/Line 44-49)** |
| 4 | Objectives | Therefore, this study was aimed to investigate antitumor-promoting activity of annonacin via *in vivo* two-stage mouse skin tumorigenesis model and its molecular pathways involved.  **(Abstract/ Page2/ Line 5-7)** |
| **METHODS** | | |
| 5 | Ethical statement | Ethical approval was obtained from Institutional Animal Care and Use Committee, Universiti Putra Malaysia prior to execution of the experiment. The reference number was UPM/IACUC/AUP-R068/2014 (Approval date: 14 January 2015).  **(Methods/ Experimental animals/ Page 6/ Line 105-108)** |
| 6 | Study design   1. Number of experimental and control 2. Steps taken to minimize bias 3. Experimental unit 4. Flow chart | 50 female ICR mice purchased from local supplier (Sapphire Enterprise, Malaysia) with age between 6-7 weeks were used in this experiment. **(Methods/ Experimental animals/ Page 6/ Line 104-105; Methods/ Anti-tumor promoting effects of annonacin (85nM) in two-stage mouse skin tumorigenesis model/ Page 7/ Line 121-139)**  Mice were randomly separated into 5 groups (n = 10) in plastic cages contained wood shaving and fed with free access standard laboratory diet (food pellet and water) *ad libitum*. **(Methods/ Experimental animals/ Page 6/ Line 110-112)**  NA |
| 7 | Experimental procedure   1. How 2. When 3. Where 4. Why | The experimental design was adapted from Abel et al. 2009 [14] as follows:  Group I (vehicle control): Mice were given topical application acetone (100 µl/mouse) on the shaved dorsal skin area twice weekly, throughout the experiment period.  Group II (carcinogen control): Mice were given single topical application of DMBA in acetone (390 nmol/100 µl/mouse), followed by topical application of TPA in acetone (1.7 nmol/100 µL/mouse) a week after DMBA application, twice weekly for 22 weeks of promotion period.  Group III (annonacin treatment): A week after DMBA single dose application, mice in this group were repeatedly promoted with TPA twice weekly for 22 weeks. 30 minutes prior to TPA application, mice were also applied topically with annonacin (85 nM/100 uL/mouse) twice weekly for 22 weeks.  Group IV (treatment control): Mice were given only annonacin (85 nM/100 uL/mouse) twice weekly for 22 weeks without DMBA initiation and TPA promotion.  Group V (reference control): Mice were treated as in group III, except the animals received the topical application of curcumin (10 mg/kg/100 µL/mouse) instead of annonacin twice weekly for 22 weeks.  **(Methods/ Anti-tumor promoting effects of annonacin (85nM) in two-stage mouse skin tumorigenesis model/ Page 7/ Line 121-139)**  All treatments were carried out at Animal Housing Facility and Pharmacology Laboratory, Faculty of Medicine and Health Sciences, Universiti Putra Malaysia. **(Methods/ Experimental animals/ Page 7/ Line 114-115).**  Topical route of administration and doses used were used as suggested in the established protocol [3]. |
| 8 | Experimental animals   1. Strain, sex, age, weight 2. Source of animal, acclimatization | 50 female ICR mice purchased from local supplier (Sapphire Enterprise, Malaysia) with age between 6-7 weeks were used in this experiment. **(Methods/ Experimental animals/ Page 6/ Line 104-105)**  ICR mice with initial weight between 20-30 g were used throughout the experiment. **(Methods/ Experimental animals/ Page 6/ Line 108-109)**  50 female ICR mice purchased from local supplier (Sapphire Enterprise, Malaysia) with age between 6-7 weeks were used in this experiment. **(Methods/ Experimental animals/ Page 6/ Line 104-105).**  Acclimatization is done to ensure all mice were healthy and free from any microbial infections, as well as to adapt with new environment provided. Acclimatization was allowed for one week prior to commencement of the experiment. **(Methods/ Experimental animals/ Page 7/ Line 113-114).** |
| 9 | Housing and husbandry   1. Housing 2. Husbandry condition 3. Welfare-related assessment | Mice were randomly separated into 5 groups (n = 10) in plastic cages contained wood shaving and fed with free access standard laboratory diet (food pellet and water) *ad libitum*. **(Methods/ Experimental animals/ Page 6/ Line 110-112)**  NA, due to only non-invasive drug administration (topical application) method is used. |
| 10 | Sample size | Mice were randomly separated into 5 groups (n = 10) in plastic cages contained wood shaving and fed with free access standard laboratory diet (food pellet and water) *ad libitum*.  **(Methods/ Experimental animals/ Page 6/ Line 110-112)** |
| 11 | Allocating animal to experimental groups | Mice were randomly separated into 5 groups (n = 10) in plastic cages contained wood shaving and fed with free access standard laboratory diet (food pellet and water) *ad libitum*. **(Methods/ Experimental animals/ Page 6/ Line 110-112)**  The experimental design was adapted from Abel et al. 2009 [14] as follows:  Group I (vehicle control): Mice were given topical application acetone (100 µl/mouse) on the shaved dorsal skin area twice weekly, throughout the experiment period.  Group II (carcinogen control): Mice were given single topical application of DMBA in acetone (390 nmol/100 µl/mouse), followed by topical application of TPA in acetone (1.7 nmol/100 µL/mouse) a week after DMBA application, twice weekly for 22 weeks of promotion period.  Group III (annonacin treatment): A week after DMBA single dose application, mice in this group were repeatedly promoted with TPA twice weekly for 22 weeks. 30 minutes prior to TPA application, mice were also applied topically with annonacin (85 nM/100 uL/mouse) twice weekly for 22 weeks.  Group IV (treatment control): Mice were given only annonacin (85 nM/100 uL/mouse) twice weekly for 22 weeks without DMBA initiation and TPA promotion.  Group V (reference control): Mice were treated as in group III, except the animals received the topical application of curcumin (10 mg/kg/100 µL/mouse) instead of annonacin twice weekly for 22 weeks.  **(Methods/ Anti-tumor promoting effects of annonacin (85nM) in two-stage mouse skin tumorigenesis model/ Page 7/ Line 121-139)** |
| 12 | Experimental outcomes | During the period of induction and treatment, each mouse in all groups of promotion stage was weighed and shaved weekly for an easy application of the carcinogens/test compound and skin lesion observation. Latency of tumor formation, percentage of tumor incidence, value of tumor burden and tumor volume were observed, measured and recorded at weekly interval. Tumors with a diameter greater than 1 mm that persists for at least 2 consecutive observations were included in the cumulative counts. The data expressed as (i) the percentage of tumor incidence ([number of mice with tumor/total number of mice] x100%) [14]; (ii) tumor burden (the total number of tumors per tumor-bearing mice) and; (iii) the tumor volume (mm³) = π/6 x length x width x height [15]. Whilst, (iv) the latency period of tumor formation was determined by the appearance of the first tumor. **(Method/Morphological assessment/ Page 8/ Line 142-151).**  All mice were sacrificed upon termination at week 22 of the experiment by cervical dislocation and sampled for further analysis. Treated mice skin area (with or without tumor) were harvested. Part of harvested skin was preserved in RNA*later* (Life Science, USA) solution for protein expression analysis. Whereas, the remaining specimens were preserved in 10% (v/v) neutral buffered formalin for standard histopathological analysis. The slides were evaluated by pathologist for assessment of the pathological changes and digital micrographs of the slides were taken using Dino-Lite microscope eyepiece camera (ANMO, Taiwan). **(Method/Histopathological assessment/ Page 8/ Line 154-160).**  Gene and protein extraction for molecular studies  **(Method/ RNA and protein extraction/ Page 9/ Line 163-172)** |
| 13 | Statistical methods | Statistical analysis was performed using SPSS version 21.0 for Windows (IBM, USA). Data were expressed as mean ± standard error of mean (S.E.M). On the other hand, the statistical difference of tumor incidence in *in vivo* data was evaluated by Chi square test. Whereas, the mean difference of tumor volume, tumor regression and tumor burden as well as molecular expression data between groups were analyzed with one-way ANOVA followed by post hoc test using Least Significant Difference (LSD). Fold-change ratio obtained from gene expression data was considered significantly upregulated and downregulated when the value is more than 2.0 and less than 0.5 fold respectively [17]. All data were considered statistically significant at *p*<0.05.  **(Method/ statistical analysis/ Page 12/ Line 242-249)** |
| **RESULTS** | | |
| 14 | Baseline data | NA. All experimental animals were survived until at the end of experiment (Please refer to Table 1 in table file) |
| 15 | Number analysed   1. Number of animals in each group included in each analysis. | (Please refer to Table 1 in table file) |
| 16 | Outcomes and estimation | (Please refer to Table 1, 2, 3 and 4 in table file) |
| 17 | Adverse events | NA. |
| **DISCUSSION** | | |
| 18 | Interpretation/scientific implication   1. Result interpretation, achievement of objective and hypothesis 2. Study limitation 3. Implication of experimental method in 3R | Collectively, the current findings provide new insight of annonacin’s mechanism of action in suppressing tumorigenesis. Annonacin demonstrates down-regulation of AKT, ERK, MTOR, p38 and Src, whilst simultaneously prevents PTEN down-regulation at gene and protein levels. Modulation of these genes eventually promotes cell apoptosis in the skin tumor cells via inhibition of PI3K/AKT/MTOR signaling pathway *in vivo*. This is evidenced by delayed papilloma development, incidence as well as its reduction of volume. On the other hand, annonacin was found to be non-toxic to liver and kidney and was able to protect skin from further development of neoplastic changes featured by absence of mitotic activity, mild hyperplasia and mild hyperkeratosis. Finally, this study suggests that annonacin may serve as a potential therapeutic compound for the prevention and treatment of skin cancer. **(Conclusion/ Page 23/ Line 568-577)**  Regimen used (DMBA 390nM/TPA 1.7 nM) was not able to induce squamous cell carcinoma, we only able to produce high grade papilloma. This might be overcome by increasing the duration of cancer induction time more than 22 weeks **(Discussion/ Page 20/ Line 422-425)**  NA |
| 19 | Generalisability/ translation to human | Topical application of annonacin could be a new potential method of treating localized skin cancer in human as evidenced through this model.  Why [3]:-   1. This mouse model allows, that tumor development can be conveniently monitored visually throughout the life span of the mice. 2. This model recapitulates features of multistage carcinogenesis in humans, tumor response in this model is highly reproducible, therefore the efficacy of chemopreventive agents can be assessed and measured successfully. 3. This model also is a good model for human cancers because humans are typically exposed to multiple low doses of both carcinogens and promoting agents. |
| 20 | Funding | The authors would like to express their greatest appreciation to Ministry of Higher Education, Malaysia for funding the work provided; a (Grant number: 04-02-11-1388RU) and Universiti Putra Malaysia- Putra Grant (GP-IPS/2015/9461000).  (Declaration/ Funding/ Page 29/ Line 625-627). |
